# Manipulating the light-matter interactions in plasmonic nanocavities at 1 nm spatial resolution

**DOI:** 10.1038/s41377-022-00918-1

**Published:** 2022-07-26

**Authors:** Bao-Ying Wen, Jing-Yu Wang, Tai-Long Shen, Zhen-Wei Zhu, Peng-Cheng Guan, Jia-Sheng Lin, Wei Peng, Wei-Wei Cai, Huaizhou Jin, Qing-Chi Xu, Zhi-Lin Yang, Zhong-Qun Tian, Jian-Feng Li

**Affiliations:** 1 Department of Physics, State Key Laboratory of Physical Chemistry of Solid Surfaces, College of Chemistry and Chemical Engineering, Xiamen University, Xiamen, 361005 China; 2 Innovation Laboratory for Sciences and Technologies of Energy Materials of Fujian Province (IKKEM), Xiamen, 361005 China; 3 College of Optical and Electronic Technology, Jiliang University, Hangzhou, 310018 China

**Keywords:** Optical spectroscopy, Nanophotonics and plasmonics

## Abstract

The light-matter interaction between plasmonic nanocavity and exciton at the sub-diffraction limit is a central research field in nanophotonics. Here, we demonstrated the vertical distribution of the light-matter interactions at ~1 nm spatial resolution by coupling A excitons of MoS_2_ and gap-mode plasmonic nanocavities. Moreover, we observed the significant photoluminescence (PL) enhancement factor reaching up to 2800 times, which is attributed to the Purcell effect and large local density of states in gap-mode plasmonic nanocavities. Meanwhile, the theoretical calculations are well reproduced and support the experimental results.

## Abstract (web summary)

The plasmon-exciton interaction between gap-mode nanocavities and A excitons of MoS2 is investigated, and the longitudinal distribution of the plasmon-exciton interactions is obtained at ~1 nm spatial resolution.

## Graphical abstract

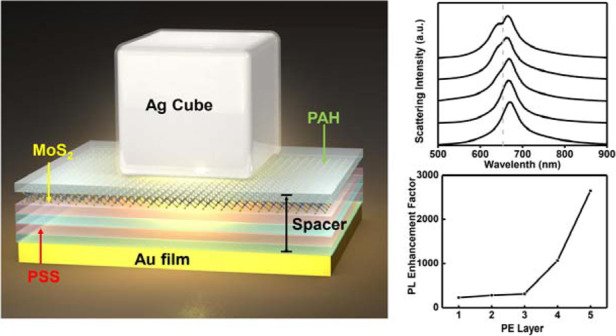

## Introduction

As a critical phenomenon for investigating fundamentals in plasmonics^1–4^, the light-matter interaction in plasmonic nanocavities at nanoscales has attracted considerable attention^5–7^. Gap-mode plasmonic nanocavity has excellent tunability for the plasmon resonance frequency or the “hot spot” position by adjusting the size, separation, or shape of system structure^8,9^, which can conveniently achieve the maximum overlap of spectral between the plasmon and emitters. A typical light-matter interaction, localized surface plasmon resonance, can generate a strong non-uniform electromagnetic field, resulting in many different phenomena, such as surface-enhanced Raman scattering^10–12^, enhanced fluorescence^13–15^, and strong coupling^16–19^.

Compared with the exchange rate and the loss rate of energy in a system, the light–matter interaction is usually divided into weak and strong coupling regimes in different nanocavities. In the former regime, the electromagnetic field in plasmonic nanocavities significantly changes the spontaneous emission rate of the system, which is called the Purcell effect^20,21^. In the latter, the rate of energy exchange between light-matter interactions exceeds damping rates in nanocavities, giving rise to new hybrid states with large Rabi splitting^22^. Meanwhile, when the system regime lies between the two, the high luminescence system can be obtained^23–25^, which means the essentiality of distinguishing different coupling regions for understanding light-matter interactions at nanoscales.

Recently, a widespread interest focused on the nanoscale spatial distributions of light-matter interaction whose coupling modes mainly depend on two system compositions’ characteristics: optical cavities and emitters. On the one hand, light-matter interactions with horizontal spatial resolution have been extensively studied. As useful techniques, the scanning antenna microscope^26^ and the scanning tunneling microscopy (STM)^27^ could precisely control the horizontal distance between the nanocavity and emitters, demonstrating the system coupling in sub-nanometer resolution. On the other hand, in studies of the light-matter interaction with vertical spatial distributions, gap-mode plasmonic nanocavity secures an advantage of proper mode volume. It constructs a concrete placement of emitters to manipulate the light at the nanoscale and respond quickly to the changes in the system. Through embedding emitters into the gap of plasmonic nanocavity, such as molecules^28^, quantum dots^29^, or transition metal dichalcogenides (TMDs)^30–32^, the desired configuration for manipulating light-matter interactions can be built. However, few studies on the vertical distribution of the actual system coupling in plasmonic nanocavities.

Here, we demonstrated a system realizing the vertical distribution of plasmon-exciton coupling at the nanoscale. The system manipulates the coupling between the A excitons of monolayer MoS_2_ and the plasmon in a gap-mode plasmonic nanocavity. More importantly, the different vertical distributions of coupling strength in the system could be accurately detected down to the nanometer scale by embedding MoS_2_ into different vertical positions. In addition, we also achieved high photoluminescence (PL) enhancement factor reaching up to 2800 times in this structure due to the formation of a highly luminescent system.

## Results

### The construction of plasmon-exciton system

**Fig. 1:**
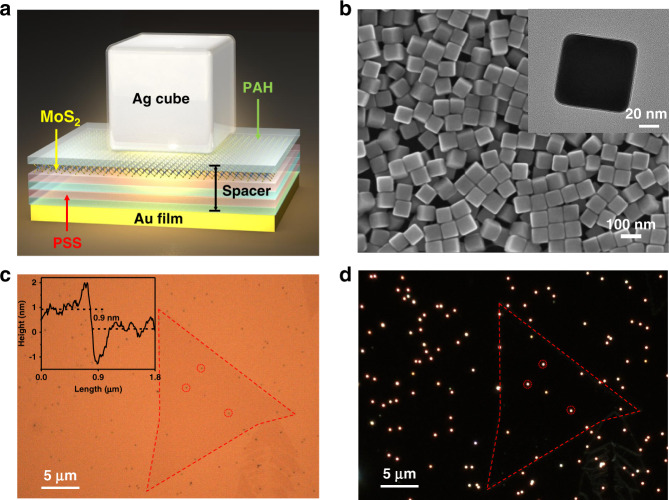
The characterization of gap-mode plasmonic nanocavity. **a** The schematic diagram of the gap-mode nanocavity, which consists of a Au film, a Ag nanocube, a monolayer MoS_2_, and PE spacer layers. **b** Scanning electron microscope (SEM) image of Ag nanocubes with 80 ± 3 nm (scale bar 100 nm). The inset is a transmission electron microscope (TEM) image of an Ag nanocube(scale bar 20 nm). **c** Bright-field image and **d** the corresponding dark-field scattering image of monolayer MoS_2_ with gap-mode nanocavities (scale bar 5 μm). The inset is the AFM height profile of monolayer MoS_2_ on PE-coated Au film

The gap-mode plasmonic nanocavities were built to optimize spatial and spectral overlap between A excitons of MoS_2_ and plasmon in nanocavity. The systems consist of an Ag nanocube over an ultrasmooth Au film, separated by polymer electrolyte (PE) layers and a monolayer MoS_2_. A schematic diagram of the nanostructure is depicted in Fig. 1a. The ultrasmooth Au film with a roughness of ~0.5 nm was prepared first, followed by the PE deposited on Au film with several nanometers as spacer layers. Then, the monolayer MoS_2_ was transferred to the top of PE layers. Subsequently, one additional PE layer was deposited as an adhesion layer to assemble the Ag nanocubes better. Finally, Ag nanocubes with an average size of 80 ± 3 nm were dropped onto the adhesion layer and dried with purity nitrogen. Figure 1b shows scanning electron microscopic (SEM) and transmission electron microscopic (TEM) images of Ag nanocube with an average size of 80 ± 3 nm, coated by 2–3 nm polyvinylpyrrolidone (PVP). Respectively, typically bright and dark-field optical images of the gap-mode nanocavities structure are shown in Fig. 1c, d, where the triangular flake represents the monolayer MoS_2_; black dots in the bright-field image and bright red dots in the dark-field image indicate the Ag nanocubes, respectively. The measured samples are labeled in Fig. 1c, d.

### The study of plasmon-exciton system with the different thicknesses of PE layers

**Fig. 2:**
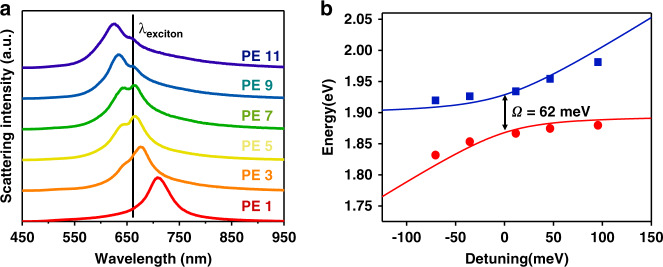
The dark-field scattering spectrums study of plasmon-exciton system with different thicknesses of PE layers. **a** Dark-field scattering spectrums of above gap-mode nanocavity with various thicknesses of PE layers. **b** The energy of the upper branch hybrid state energy (red) and the lower branch hybrid state energy (blue) as a function of detuning

Atomically thin monolayer MoS_2_ has large exciton binding energy and exciton radius at room temperature, which is caused by the reduction of dielectric scattering and the limitation of carriers^33^. And the direct band gap transition of monolayer MoS_2_ is dominated by the A excitons peak (~656 nm). Therefore, MoS_2_ can be well combined with plasmon gap modes, one can build up an ideal platform for studying plasmon-exciton interaction and related applications. By adjusting the number of PE layers to obtain different thicknesses (Table 1), we can easily obtain various plasmon resonance frequencies by dark-field scattering spectrums. A self-made confocal dark-field microscope is used to characterize the plasmon resonance of different nanocavities with various thicknesses of PE layers, as shown in Fig. 2a. With the increase of PE layers thickness, the plasmon resonance frequencies gradually blue-shifts across the A exciton peak at 656 nm in Fig. S1. Meanwhile, the normalized dark-field scattering spectrums show peaks splitting with a characteristic dip at the wavelength of the A excitons (black line), which indicates the coherent coupling with plasmon-exciton interaction. When the materials of the Spacer layer are replaced by the air, there is no Rabi splitting in the scattering spectrums compared with the current Spacer (Fig. S12).

**Table 1:** Thickness of PE spacer with different layers (measured by Ellipsometry)

Sample	1-PE	3-PE	5-PE	7-PE	9-PE	11-PE
Thickness	1.0 nm	2.9 nm	5.5 nm	8.4 nm	11.5 nm	14.8 nm

Since the system involves the participation of multiple excitons, we used the classical coupled harmonic oscillator model (CHOM) to describe the process of plasmon-exciton interaction. In this structure, the excitons involved are described as "super oscillators", so the system can be simply described as two coupled oscillators^34^:

1 $$\left( {\begin{array}{*{20}{c}} {{\it\mathrm{E}}_{{\it\mathrm{sp}}} - {\it\mathrm{i}}{{\Gamma }}_{{\it\mathrm{sp}}}/2} & {\it\mathrm{g}} \\ {\it\mathrm{g}} & {{\it\mathrm{E}}_{{\it\mathrm{ex}}} - {\it\mathrm{i}}{{\Gamma }}_{{\it\mathrm{ex}}}/2} \end{array}} \right)\left( {\begin{array}{*{20}{c}} \alpha \\ \beta \end{array}} \right) = {{{\mathrm{E}}}}\left( {\begin{array}{*{20}{c}} \alpha \\ \beta \end{array}} \right)$$

where E_sp_ and E_ex_ are the energy of the plasmon in nanocavity and the A exciton in monolayer MoS_2_; g is the coupling strength of the system; Γ_sp_ and Γ_ex_ represent the dissipation rate of plasmons and excitons, respectively; E represents the eigenvalues corresponding to the energies of the new plexcitons; α and β are the eigenvector components. Solving the Eq. (1) and assuming the widths of exciton and plasmon are small compared to their energies and neglecting the high order parts in the widths of exciton and plasmon, we can get the solution Eq. (2):

2 $${{{\mathrm{E}}}}_ \pm = \frac{1}{2}\left( {{{{\mathrm{E}}}}_{{{{\mathrm{sp}}}}} + {{{\mathrm{E}}}}_{{{{\mathrm{ex}}}}}} \right) \pm \sqrt {{{{\mathrm{g}}}}^2 + \frac{1}{4}\delta ^2}$$

here, δ = E_sp_–E_ex_ represents the detuning energy between the A exciton of monolayer MoS_2_ and plasmon in nanocavity. The result about matching of the detuning data and the numbers of PE layers is shown in Fig. S2.

The scattering spectrum in the two-oscillator system can be expressed as^35–38^:

3 $$\sigma _{\mathrm{scat}}\left( {\mathrm{E}} \right) = {\mathrm{AE}}^4\left| {\frac{({\mathrm{E}}^2 - {\mathrm{E}}_{{\mathrm{ex}}}^2 + {\mathrm{iE}}{{\Gamma }}_{{\mathrm{ex}}})}{{\left( {{\mathrm{E}}^2 - {\mathrm{E}}_{\mathrm{ex}}^2 + {\mathrm{iE}}{{\Gamma }}_{\mathrm{ex}}} \right)\left( {{\mathrm{E}}^2 - {\mathrm{E}}_{\mathrm{sp}}^2 + {\mathrm{iE}}{{\Gamma }}_{\mathrm{sp}}} \right) - 4{\mathrm{E}}^2{\mathrm{g}}^2}}} \right|^2$$

where A represents the scattering amplitude. To extract the coupling strength, we use Eq. (3) to fit the scattering spectra in Fig. 2a, as shown in Fig. S3. It is noted that fitting results also show an anticrossing behavior.

To map the dispersion curve, we extract the two peaks of multiple scattering spectra in Fig. 2a, as marked in Fig. 2b (blue and red dots). Anticrossing fitting results are shown in Fig. 2b (solid curves), which agree well with the experimental data. The plasmon line width of the system can be extracted to be ~160 meV from Fig. S1, while the line width of MoS_2_ monolayers is extracted to be ~70 meV from Fig. S4. When detuning δ = 0, we extract the vacuum Rabi splitting $\Omega = 2{{{\mathrm{g}}}} = 62 meV$. The Rabi splitting obtained here is smaller than the line width of plasmons (160 meV) but close to excitons (70 meV), and the scattering spectrums of the hybrid system show a Rabi splitting, which indicates the coupling strength of the gap-mode system is in the transition region between weak and strong coupling region. Fig. S3 shows the corresponding coupling strength as a function of the spacer thickness. It is noted that the coupling strength is weak when the PE layer thickness is small, and gradually increases and stabilizes as the PE layer thickness increases. This is due to the fact that when the PE layer thickness is small, the resonance peak position of the plasmon nanocavity is far away from the exciton absorption peak, so the coupling between them is weak. And we also measured the PL intensity with different PE layers in these gap-mode nanocavities and found that the PL enhancement factor of the five PE layers was the largest, which was very similar to previous reports (Fig. S6).

### The study of plasmon-exciton system with the same thickness of PE layers at 1 nm spatial resolution

**Fig. 3:**
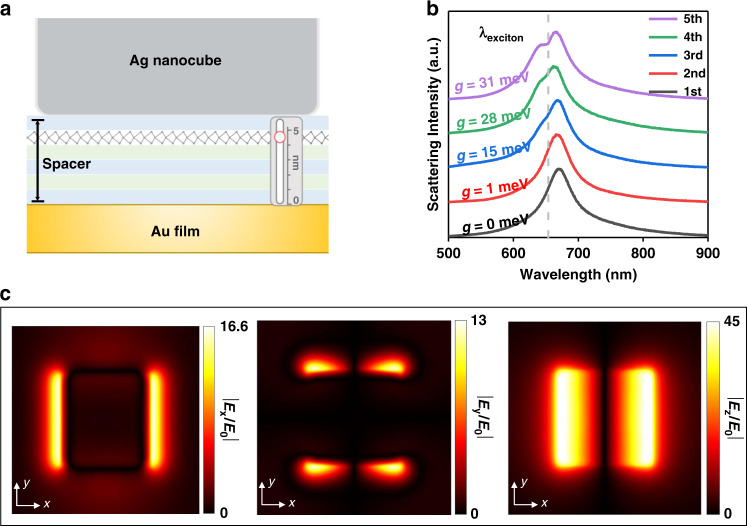
The dark-field scattering spectrums study and theoretically calculation for electromagnetic field distribution of plasmon-exciton system with the same thickness PE layers. **a** Schematic diagram of MoS_2_ at the above nanocavities with the thickness of 5 PE layers. **b** The dark-field spectrum of nanocavities was measured with different positions of MoS_2_. **c** Distribution of the x, y, z-component of the electric field in the XY planes situated at 5th position in Fig. 3a with MoS_2_ is background index-only material, normalized to the incident electric field

To further clarify the plasmon-exciton interaction in the nanocavity, we conducted more detailed research on the basis of the model. We fixed the height of spacer layers to 5 PE layers and adjusted the position of MoS_2_ in nanocavities by changing the order among MoS_2_ transferred and PE deposited. To assemble the Ag nanocubes better, we always keep the poly(allylamine) hydrochloride (PAH) on the top of spacer layers, as shown in Fig. 3a. Thus, we obtain different dark-field scattering spectrums at ~1 nm spatial resolution (Fig. 3b). When MoS_2_ is located at the bottom of the PE layer, the dark-field scattering spectrum shows an ordinary Lorentz-shaped peak; When MoS_2_ approaches the Ag nanocube, the scattering spectra gradually split, which also proves that the plasmon-exciton coupling strength is different at different positions in the nanocavity. Fitting parameters g_i_ (*i* = 1, 2, 3, 4, 5) equal to 0, 1, 15, 28, and 31 meV by using the coupled oscillator model, respectively (see Fig. S8).

The difference in dark-field splitting degree when MoS_2_ is located at different positions in nanocavities implies the different coherent interaction strength between excitons and plasmons in the nanocavity^39,40^. The following points can explaine the phenomenon. As shown in Fig. S7, the electric field of the nanocavity with MoS_2_, which is background index-only material, is unevenly distributed. The total coupling strength g is the summation of the coupling strength for individual exciton g_0_(r), where ${{{\mathrm{g}}}}_0\left( {{{\mathbf{r}}}} \right) = {\upmu}{{{\mathbf{E}}}}\left( {{{\mathbf{r}}}} \right)$, μ is the vacuum magnetic permeability and ***E*** is the in-plane vacuum electric field. We can find the vacuum electric field is highly spatial dependent on the nanocavity from Fig. 3c. and Fig. S7. Therefore, when MoS_2_ approaches the Ag nanocubes, the electric field intensity behaves higher at the location of excitons. As a result, the coupling strength reaches the maximum value as immense as 31 meV. In addition, a part of the XY plane electric field will be generated near the nanocube due to the certain curvature of the edge of the nanocube. Therefore, the electromagnetic field and the MoS_2_ exciton dipole moment can produce stronger coupling.

On the other hand, a certain amount of energy may be transferred between MoS_2_ and the substrate when the MoS_2_ approaches the Au film. Hence, the energy dissipation rate increases, leading to a remarkable reduction of the coupling strength. In plasmonic nanocavities, excitons coupled with plasmon efficiently radiate with a high radiative rate and directivity. With considerable spatial overlap in sub-wavelength mode volume, the coupling strength affects the luminous ability of the system. The formation of plexcitons usually shows excellent luminous ability. Thus, the relationship between plasmon-exciton systems with different coupling strengths and photoluminescence attracts attention for its significance in applications. Therefore, we performed corresponding PL spectrum acquisition in the gap-mode nanocavities in Fig. 3.

### The PL spectrum of plasmon-exciton system with the same thickness of PE layers

**Fig. 4:**
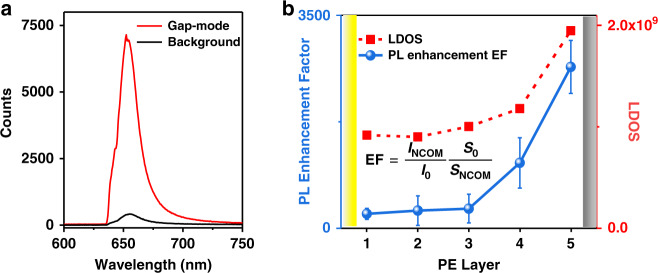
The PL spectrum study of monolayer MoS_2_ at the above gap-mode nanocavity with the same thickness of PE layers. **a** PL spectra of monolayer MoS_2_ with (red) and without (black) gap-mode nanocavity. **b** The blue line is the enhancement factor of the PL at different positions; the red line is the LDOS at different positions

We use the 633 nm laser to excite the sample and collect the PL signal of the sample. Figure 4a shows the PL spectrum of MoS_2_ in the top layer, obtaining ~23 times the spectral enhancement. It should be noted that there are some prominent peaks in the PL measured. This is due to the resonance Raman excited by the 633 nm laser, which is greatly enhanced in the plasmon nanocavity. The specific resonance Raman spectrum See Fig. S5.

In order to measure the PL enhancement of the gap-mode nanocavity, we define the PL enhancement factor (EF) as:

4 $${\it\mathrm{EF}} = \frac{{{\it\mathrm{I}}_{{\it\mathrm{NC}}}}}{{{\it\mathrm{I}}_0}}\frac{{{\it\mathrm{S}}_0}}{{{\it\mathrm{S}}_{{\it\mathrm{NC}}}}}$$

here, I_NC_ is the PL intensity of the area on MoS_2_ with Ag nanocubes, and I_0_ is the PL intensity of the area on MoS_2_ without Ag nanocubes. S_0_ is the collection area where the laser spot collection diameter is ~1 μm; and S_NC_ is the area occupied by the single-particle Ag nanocube (6400 nm^2^).

As shown by the blue line in Fig. 4b, the PL enhancement factor of each layer is calculated by Eq. (3). When MoS_2_ is located on the fifth layer closest to the Ag nanocube, the maximum enhancement factor of 2800 times is obtained, which is considerable compared with other works^41^. In addition, the PL enhancement effect shows a certain positive correlation with the coupling strength, which also confirms the influence of the strength of the plasmon-exciton interaction on the system's luminous ability.

The PL intensity of emitters is determined by their excitation rate and emission efficiency. When the plasmon resonance matches the wavelength of the pump laser, the excitation rate of emitters will be enhanced, which is proportional to near-field intensity enhancement. Therefore, the electric field enhancement in Fig. S7 reflects the excitation of emitters. However, the electric field distribution trend does not entirely conform to the PL enhancement trend. Apparently, the electric field distribution is insufficient to explain the PL enhancement trend. Additionally, because the spontaneous emission of the emitter is always determined by the local density of states (LDOS) of its photonic environment, we use LDOS to reflect the emission of emitters to explore the mechanism of the large PL enhancement^24^. We calculated in-plane LDOS of the sample with MoS_2_ excitons and with MoS_2_ that is treated as background index-only material, respectively (Fig. S9a). It is found that the presence of MoS_2_ excitons will increase the in-plane LDOS. Moreover, the Rabi splitting will cause a fast and coherent energy exchange between the emitter and nanocavity. Therefore, there is a stronger electric field inside MoS_2_ with the increase of the coupling strength. In addition, we calculated Purcell factors as a function of spacer thickness (Fig. S9b). The larger LDOS can accelerate the spontaneous emission of the emitter, so the maximum LDOS corresponds to the maximum Purcell factor. In Fig. S10, we estimated the PL lifetimes of the plasmonic NCoM cavity at different positions^21,42^. PL lifetime can be described as ${\uptau} = 1 / ({{\Gamma }} + {{\Gamma }}_{nr})$, where Γ is radiative decay rate and Γ_nr_ is nonradiative decay rate. It is noted that lifetime gradually increases as MoS_2_ approaches the Ag nanocube. When MoS_2_ is close to the Au film, the energy transfer between the two accelerates its nonradiative decay rate, which in turn reduces lifetime. Moreover, the radiation efficiency is crucial to quantify the actual PL enhancement factors of the exciton-NCoM coupling system. Fig. S11 showed the radiation efficiency (defined as ${{\Gamma }} / ({{\Gamma }} + {{\Gamma }}_{nr})$) of the plasmonic nanocavity. When MoS_2_ is located on the fifth layer closest to the Ag nanocube, the maximum radiation efficiency of the plasmonic nanocavity can be obtained. This is why the PL enhancement increases as g increases.

## Discussion

In conclusion, we investigated the plasmon-exciton interaction between gap-mode nanocavities and A excitons of monolayer MoS_2_ and realized the coupling of excitons and plasmons by using PE as spacer layers. We extracted the Rabi splitting of 62 meV from dark-field scattering spectrums of different nanocavities with various thicknesses of spacer layers, and the coupling strength was in the transition region between weak coupling and strong coupling region. In addition, we found that the coupling strength is very sensitive to the position of the excitons, which changed significantly at nanometer spatial resolution. Based on this property, we demonstrated that the coupling strength owned to the position of excitons affects the PL of the system. When the coupling strength of the system is maximum, the highest enhancement factor of 2800 times was obtained. Our results provide ideas for manipulating the interaction between excitons and plasmons in gap-mode nanocavities and open up possibilities for potential quantum optics applications.

## Materials and methods

### The synthesis of Ag nanocubes

Ag nanocubes were synthesized by a previous method^43^. Typically, 0.4 g AgNO_3_ and 0.39 mg CuCl_2_·2H_2_O were added to a 15 mL conical flask and dissolved in 10 mL 1,5-Pentanediol. 0.2 g PVP was added to another conical flask and dissolved in 10 mL 1,5-Pentanediol. The 20 mL 1,5-pentanediol was added to a round bottom flask and heated at 193 °C in the oil bath for 15 minutes. Then, the two precursor solutions in conical flasks were injected simultaneously into the round bottom flask at the rate of 0.5 mL min^−1^ with stirring. Finally, the round bottom flask was transferred to an ice bath to stop the reaction, showing that the color of the reaction solution turned deep green.

### Sample substrates fabrication

Layered MoS_2_ was synthesized by using sulfur (99.95% purity) and molybdenum oxide (MoO_3_, 99.5% purity) as precursors of S and Mo, respectively. And the growth was regulated by the following three main factors: flow rate, temperature, and hydrogen content in the carrier gas. Typically, the growth substrate was soda-lime glass, and the wafer with a polished surface oriented to the MoO_3_ powder was placed upside down on a quartz boat, then located the above quartz boat in the center of the CVD quartz tube. The temperature of this reaction was 700 °C for 20 min. The sulfur powder was placed at 250 °C upstream regions. The carrier gas and reducing atmosphere were the mixtures of Ar/H_2_ with a flow rate of 50 sccm.

Ultrasmooth Au film was prepared by a template stripping method^44^, and the Polyelectrolyte (PE) spacer layers were prepared by deposition layer-by-layer (LBL). Firstly, we dipped the Au substrates in the mixed solution of poly(allylamine) hydrochloride (PAH) and sodium chloride (NaCl) aqueous solution (1 mM PAH and 1 M NaCl) for 5 min, then washed the substrate with deionized water to obtain an Au substrate with ~1 nm thickness of PAH layer. Next, we put the above substrate in the mixed solution of polystyrene sulfonate (PSS) and NaCl aqueous solution (1 mM PSS and 1 M NaCl) for 5 min, and then washed the substrate with deionized water, so we obtained an Au substrate with 1 nm thick PSS layer deposited on the PAH surface by electrostatic interaction. After each deposition step, the substrate should be dipped in 1 mM NaCl aqueous solution to stabilize the PE layers. Alternating this process with PAH and PSS, we produced a PE film as spacer layers on Au substrate with precisely controlled thickness.

Then we pressed the PDMS stamp against the MoS_2_ on the soda-lime glass and dipped the PDMS into hot water for ~5 min to separate the MoS_2_ from the glass. After that, the PDMS stamp was peeled off with the MoS_2_ attached and was subsequently pressed against the Au film covered by the PE layer. After PDMS was peeled off, the Au film with MoS_2_ was dipped in PAH solution (1 mM PAH and 1 M NaCl) and washed with deionized water.

Finally, we deposited Ag nanocubes on the PE-coated substrates. In this part, we first washed and centrifuged 50 μL Ag nanocubes colloid with alcohol and deionized water. Then we diluted the concentration of Ag nanocubes to 1000 times with deionized water. Then, 10 μL Ag nanocubes colloid was dropped on the sample for 5 min to build up the gap-mode nanocavities with MoS_2_.

### Optical measurements

The dark-field scattering spectra of a single nanocavity on Au film were collected by a reflection dark-field microscope (Leica, upright) equipped with Renishaw in Via Raman instrument. The light source of the dark-field microscope is a 100 W halogen lamp. We collected the scattering, PL, and Raman spectrums through the same objective (NA = 0.75, ×100, Leica). After passing through a 30 μm slit, the signal was dispersed on a 150 and 1800 g/mm grating and finally collected by the CCD. The 633 nm CW laser was used to obtain PL and Raman spectra with a power of ~1.6 μW and ~7.8 μW, respectively. The integration time was set at 10 s.

## Supplementary information

Supplementary Information for Manipulating the Light-Matter Interactions in Plasmonic Nanocavities at 1 nm Spatial Resolution
