# Supplementary material for: Manipulating the light-matter interactions in plasmonic nanocavities at 1 nm spatial resolution
Source: Light Sci Appl. 2022 Jul 26;11:235. doi: 10.1038/s41377-022-00918-1 (PMC9325739; doi:10.1038/s41377-022-00918-1)
Supplement: Supplementary file 1 — Supplementary Information for Manipulating the Light-Matter Interactions in Plasmonic Nanocavities at 1 nm Spatial Resolution [file 41377_2022_918_MOESM1_ESM.docx]

Supplementary Information for

**Manipulating the Light-Matter Interactions in Plasmonic Nanocavities at 1 nm Spatial Resolution**

Bao-Ying Wen, ^1,2^ Jing-Yu Wang,^1^ Tai-Long Shen,^1,2^ Zhen-Wei Zhu,^1^ Peng-Cheng Guan,^1,2^ Jia-Sheng Lin,^1,2^ Wei Peng,^1,2^ Wei-Wei Cai,^1^ Huaizhou Jin,^1*^ Qing-Chi Xu^1*^, Zhi-Lin Yang, ^1*^ Zhong-Qun Tian, ^1,2^ and Jian-Feng Li^1,2,3*^

^1^Department of Physics, State Key Laboratory of Physical Chemistry of Solid Surfaces, College of Chemistry and Chemical Engineering, Xiamen University, Xiamen, 361005, China.

^2^Innovation Laboratory for Sciences and Technologies of Energy Materials of Fujian Province (IKKEM), Xiamen 361005, China.

^3^College of Optical and Electronic Technology, Jiliang University, Hangzhou 310018, China.

Correspondence: jinhz@xmu.edu.cn (Huaizhou Jin), Qing-Chi Xu ([xuqingchi@xmu.edu.cn](mailto:xuqingchi@xmu.edu.cn)), Zhi-Lin Yang ([zlyang@xmu.edu.cn](mailto:zlyang@xmu.edu.cn)) or Jian-Feng Li ([li@xmu.edu.cn](mailto:li@xmu.edu.cn))

These authors contributed equally: Bao-Ying Wen, Jing-Yu Wang, Tai-Long Shen.

1. **Dark field scattering spectroscopy characterization of individual gap-mode nanocavity.**

**Fig. S1.** Normalized dark field scattering spectrum of gap-mode plasmonic nanocavities with different PE layers (without MoS_2_). We used one PE layer instead of molybdenum disulfide in nanogap. Dark field scattering spectrum gradually blue shifts as the increase of PE thickness.

1. **The matching of the detuning data with the number of PE layers**

The detuning $\delta=E_{\mathrm{sp}}-E_{\mathrm{ex}}$ represented the energy between the A exciton of monolayer MoS_2_ and plasmon in nanocavity, where the value E_ex_ was defined as 1.89 eV, according to the PL data, for the value E_SP_ of each PE layer, it was calculated by E_SP_ = E_+_ + E_−_ − E_ex_. Therefore, the result about matching of the detuning data and the numbers of PE layers was shown as below.





**Fig. S2.** The matching of the detuning data and the numbers of PE layers


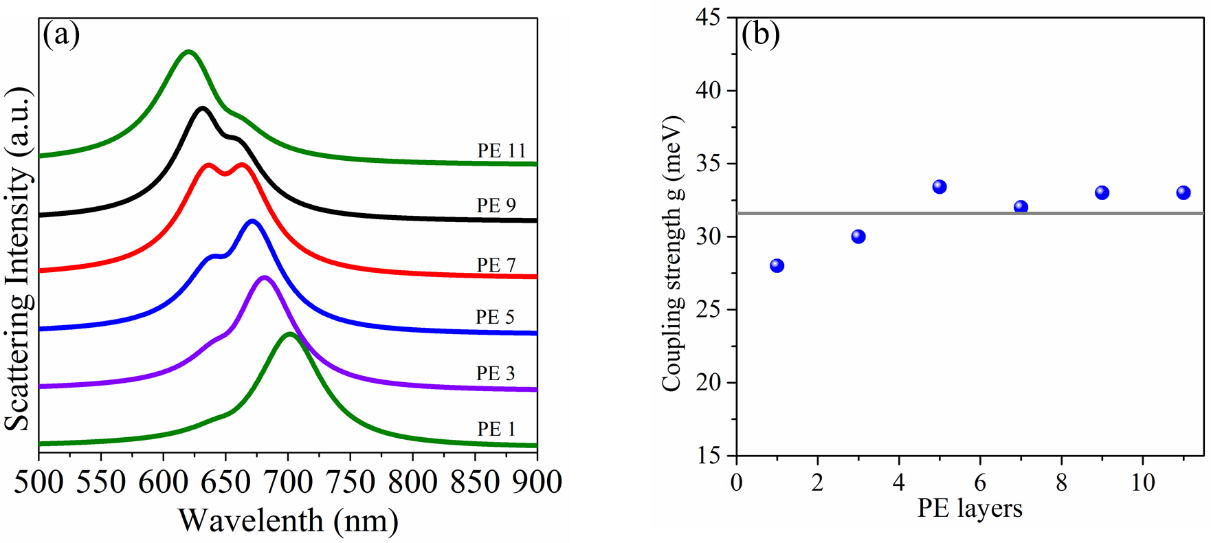


**Fig.S3.** (a) The fitting curves of nanocavities with various thicknesses of PE layers by the eq. (3). (b) Coupling strength as a function of the spacer thickness. The gray line denotes the averaged value of g.

1. **The absorption and dark-field scattering spectrum of exciton, plasmon and plasmon-exciton**


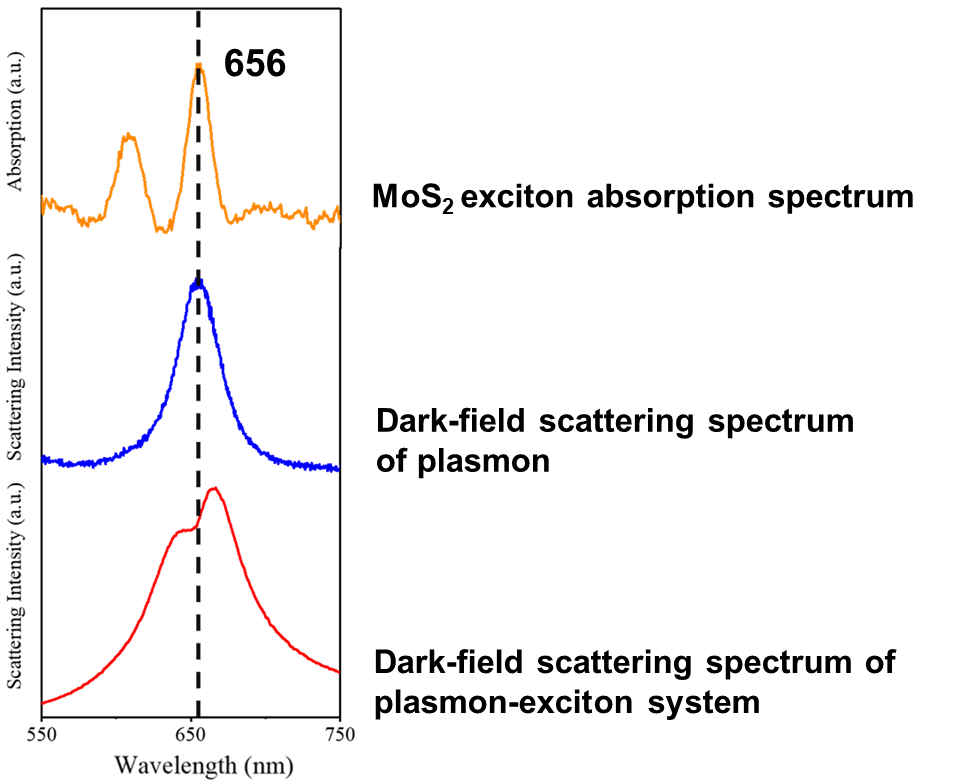


**Fig.S4** The exciton absorption spectrum of MoS_2_ and the dark-field scattering spectrum of plasmon and plasmon-exciton system, respectively.

1. **Resonant Raman scattering in monolayer MoS_2_.**


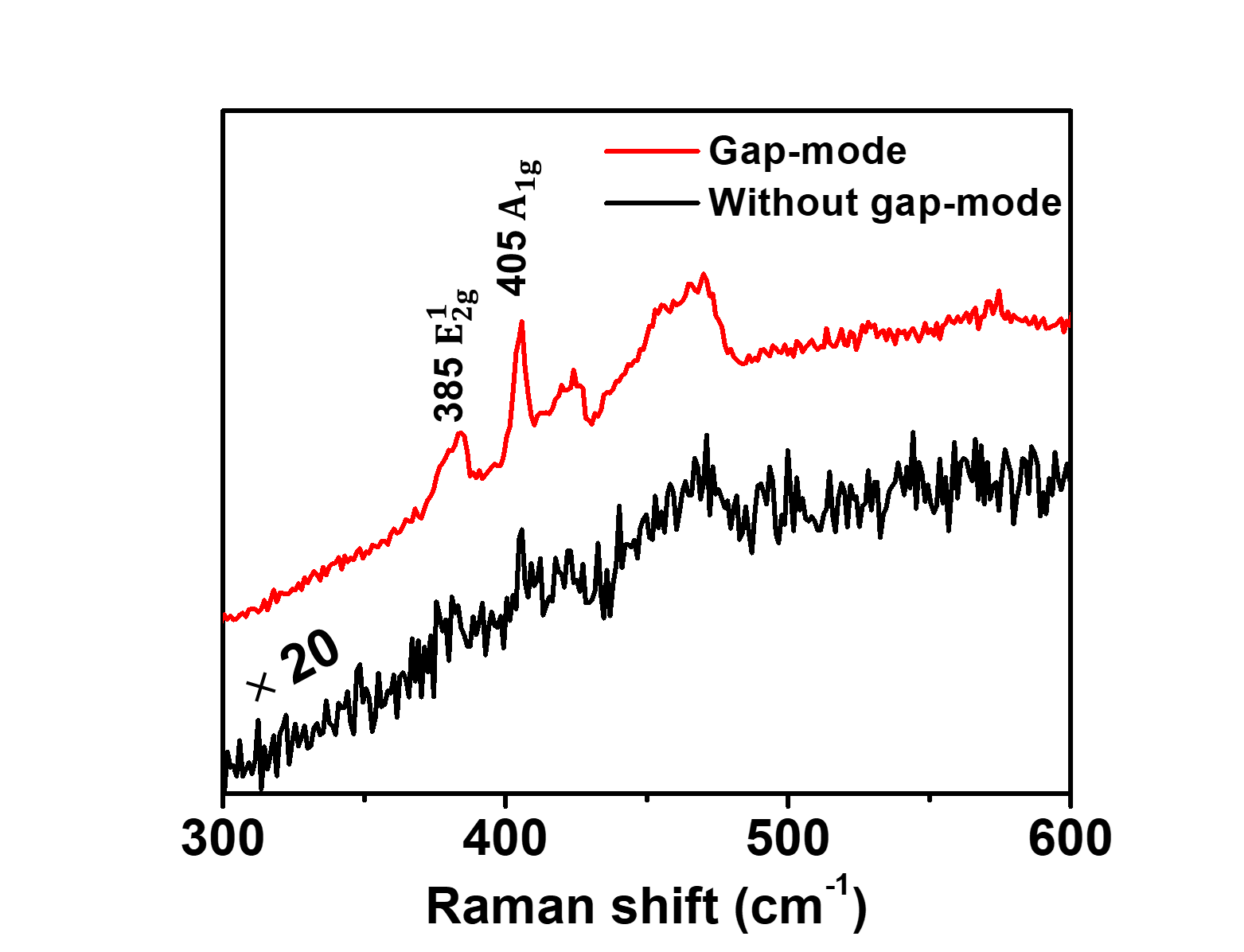


**Fig. S5.** Resonant Raman scattering spectra of monolayer MoS_2_ with gap-mode (red) and without (black) gap-mode nanocavity at same power by 633 nm laser. We can find that the characteristic Raman peak of MoS_2_, the $A_{1g}$ and $E_{2g}^{1}$ peak were at 405 cm^-1^ and 385 cm^-1^, respectively. In addition, many second-order Raman vibrations of MoS_2_ have been significantly enhanced due to the resonant Raman effect.

1. **The PL enhancement factor for samples in Fig. 2.**

**
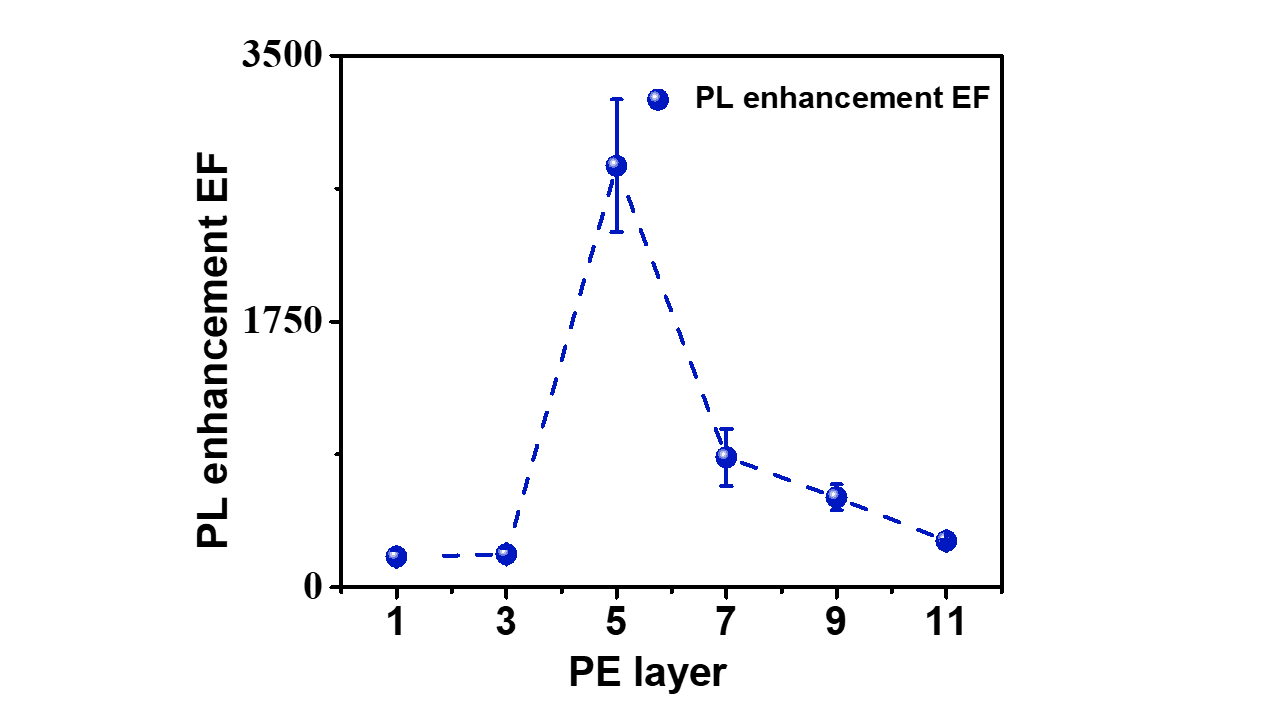
**

**Fig. S6.** The PL enhancement factor (EF) as function of PE layers. We measured the PL intensity with different PE layer in gap-mode nanocavities, and found that the PL enhancement factor of five PE layer was the largest, which was very similar to previous reports. We also attributed unusually lower enhancement factors in PE layer 1 and 3 to the quenching of the substrate.

1. **Numerical Simulations.**

Simulations were carried out by the commercial finite difference time domain (FDTD) software package (Lumerical Company). The nanoparticle was modeled as a single nanocube with a size of 80 nm and the edges are smoothed with a radius of curvature of 8 nm. The dielectric function of Ag was taken from a multicoefficient fitting model offered by Lumerical FDTD. The PVP and PE layer were modeled as dielectrics with index of refraction n = 1.4. The thickness of monolayer MoS_2_ was taken as 1 nm. The permittivity of monolayer MoS_2_ followed as a Lorentz oscillator:

$\varepsilon=\varepsilon_{0}+f_{0}\frac{\omega_{ex}^{2}}{\omega_{ex}^{2}-\omega^{2}-i{\gamma\Gamma}_{ex}\omega}$ (S1)

where ε_0_=18 is the background permittivity, *f*_0_=0.4 is the oscillator strength, ω_ex_=1.89 eV and Γ_ex_ =70 meV is the exciton line width. For background index-only material, f_0_ was set to zero.

In simulations, a total-field scattered-field source acts as a linearly polarized light normally incident on the nanostructure. Perfectly matched layer boundary conditions were adopted in order to avoid unphysical reflections around structures. The simulation time was set to 1000 fs, which sufficiently warranted the convergence. For computation-time saving, nonuniform mesh method was generated. For the narrow dielectric gap, 0.5 nm × 0.5 nm × 0.5 nm mesh size was set, whereas 1 nm × 1 nm × 1 nm was used in other regions.


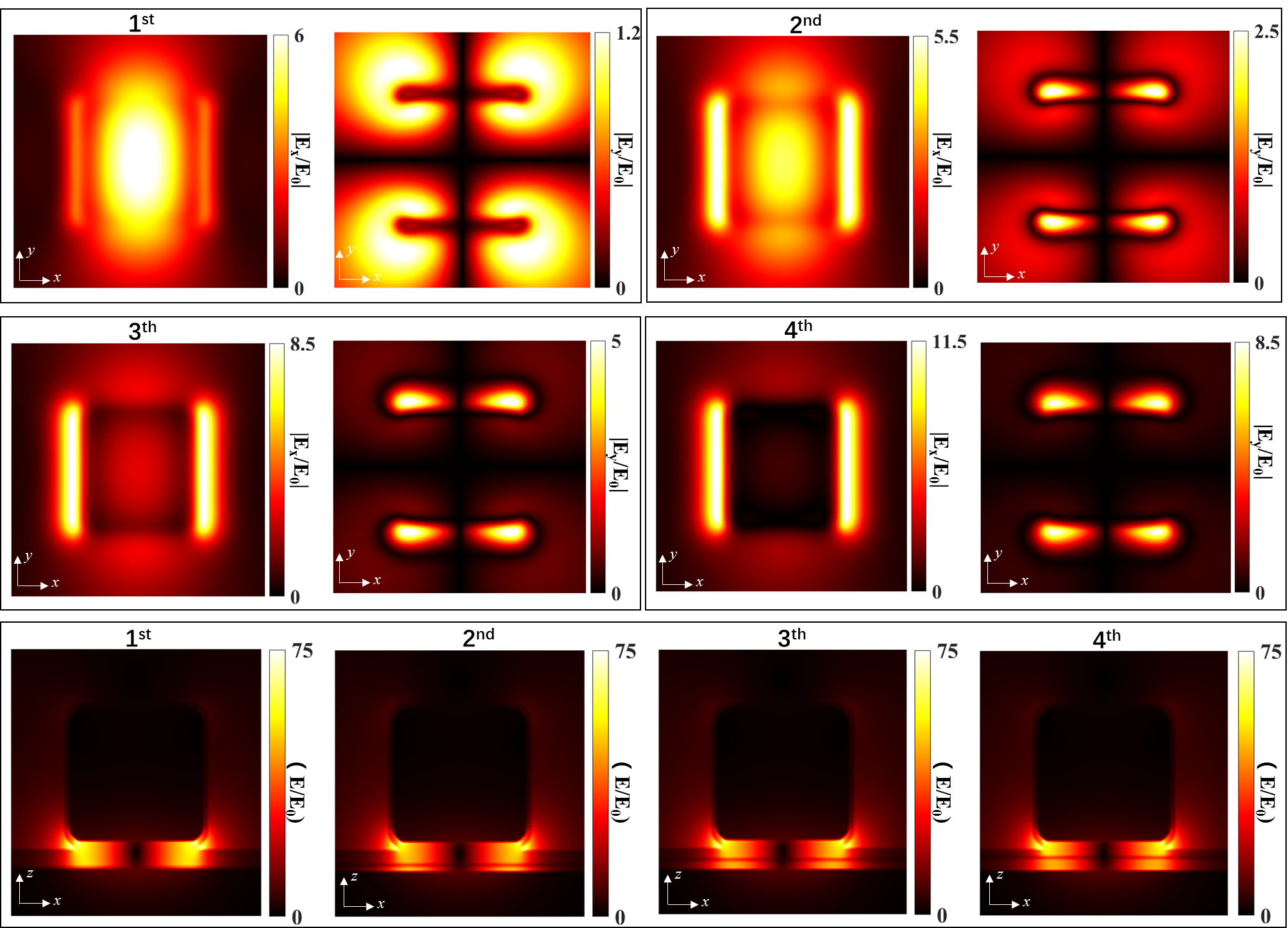


**Fig. S7.** Distribution of the x, y-component of the electric field in the XY planes at different position of Fig. 3a with MoS_2_ that is background index-only material, normalized to the incident electric field. Distribution of the electric field in the XZ planes at different position of Fig. 3a with MoS_2_ that is background index-only material.

According to the coupled oscillator model, the scattering spectrum of the hybrid system follows

$\sigma_{scat}\left( E \right)=AE^{4}\left| \frac{(E^{2}-E_{ex}^{2}+iE\Gamma_{ex})}{\left( E^{2}-E_{ex}^{2}+iE\Gamma_{ex} \right)\left( E^{2}-E_{sp}^{2}+iE\Gamma_{sp} \right)-4E^{2}g^{2}} \right|^{2}$ (S2)

where A represents the scattering amplitude, E_ex_ and E_pl_ stand for the resonance energy of the MoS_2_ exciton and the plasmon mode, respectively, while Γ_ex_ and Γ_sp_ are the dissipation terms. We fit the scattering spectra in Fig. 3b using eq (S2), as shown Fig. S8a and show the matching of the measured curves and fitting curves in Fig. S8b.


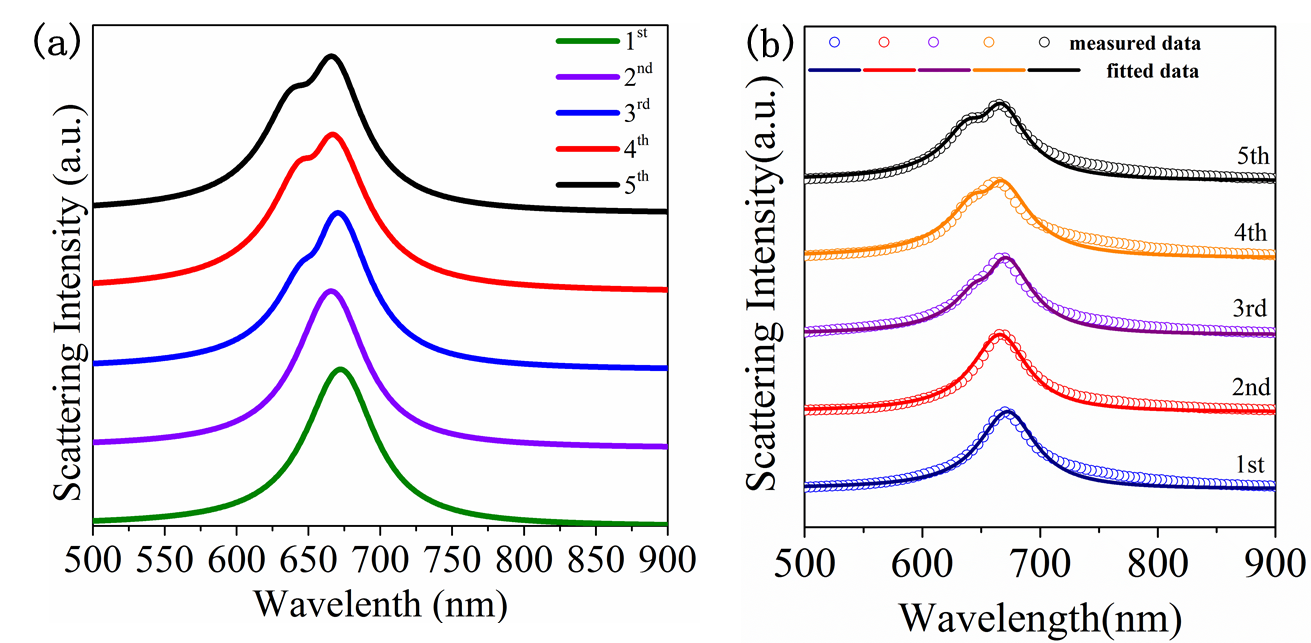


**Fig. S8.** (a) The fitting curves of nanocavities with different positions of MoS_2_ by the eq. (S2). (b) The matching of the measured curve (empty circles) and fitting curves (solid lines) of nanocavities with different positions of MoS_2._

1. **The calculation of LDOS.**


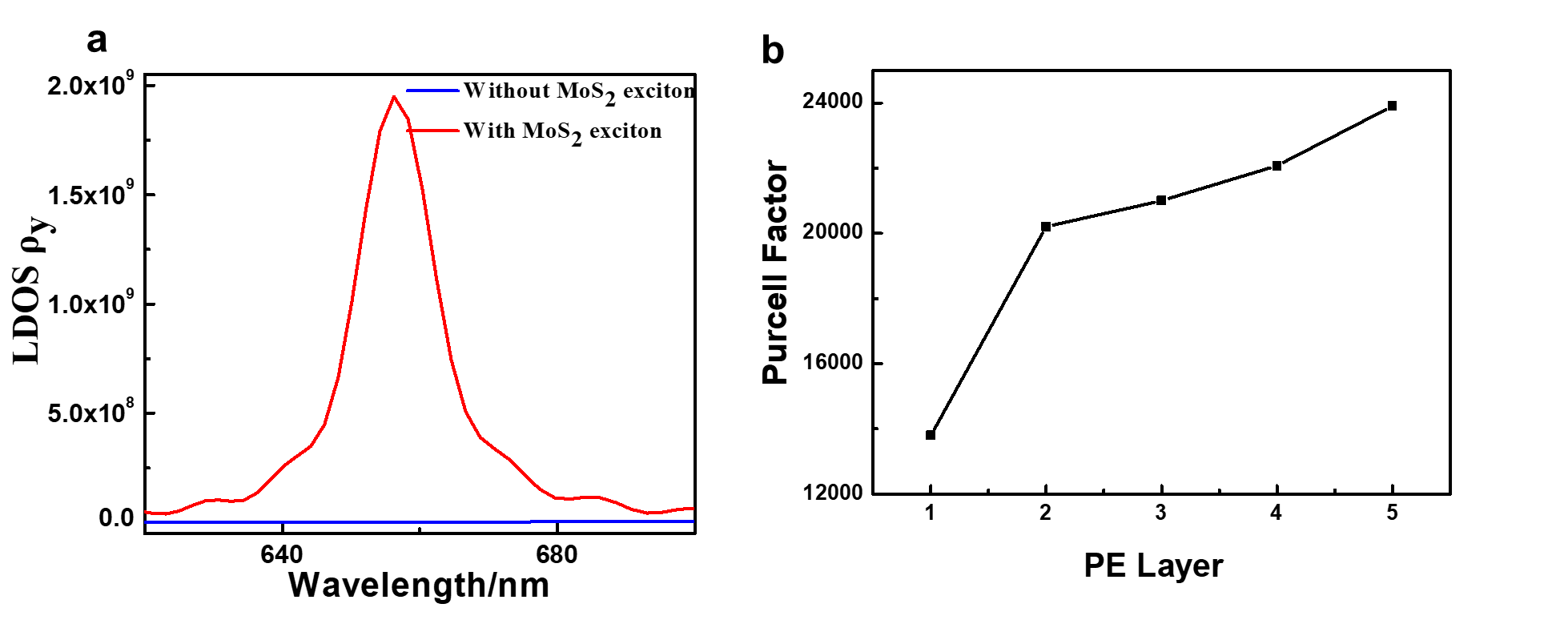


**Fig. S9.** (a) LDOS spectra of the nanostructure with (red line) and without (blue line) monolayer MoS_2_ exciton. The inset shows the top view of the nanocube with a yellow dot representing the position of a y-polarized dipole and the dipole is suited at the 5^th^ position in the Fig 3a. (b) The Purcell factors as a function of spacer thickness.

We obtained the LDOS spectra of the nanostructure with (red line) and without (blue line) monolayer MoS_2_ exciton as shown in Fig. S9^1^. The former (~10^9^) is two orders of magnitude larger than the latter (~10^7^). Furthermore, as MoS_2_ moved away from the nanocube, LDOS showed a downward trend.

The exciton of MoS_2_ was modeled as a y-polarized electric dipole source. We set the dipole at (x, y) = (35 nm, −35 nm). The dyadic Green’s function ***G*** is defined by the electric field at the dipole point ***r***:

$$\mathbf{E}\left(\mathbf{r}\right)=\frac{\omega^{2}}{\varepsilon_{0}\varepsilon_{r}c^{2}}G\left(r,r_{0}\right) \cdot\mu$$

where 𝜔 is the frequency of dipole and ***μ*** is the dipole moment. ***G*** is a 3x3 symmetric matrix and each component of ***G*** can be calculated using the corresponding dipole orientation, for example, ***G_yy_*** can be calculated from a y-polarized dipole:

$$G_{yy}=\frac{\varepsilon_{0}\varepsilon_{r}c^{2}}{\mu\omega^{2}}E_{y}$$

The local density of states along y direction can be obtained from the imaginary part of Green's function:

$$\rho_{y}\left(r_{0},\omega\right)=\frac{6\omega}{\pi c^{2}}\left[\text{Im}\left\{G_{yy}\left(r_{0},\omega\right)\right\}\right]$$

The Purcell factor of the cavity can be defined as:

$$F=\frac{3\lambda^{3}Q}{4\pi^{2}V}$$

where λ, Q, and V are the resonance wavelength, quality factor, and effective mode volume of the cavity, respectively


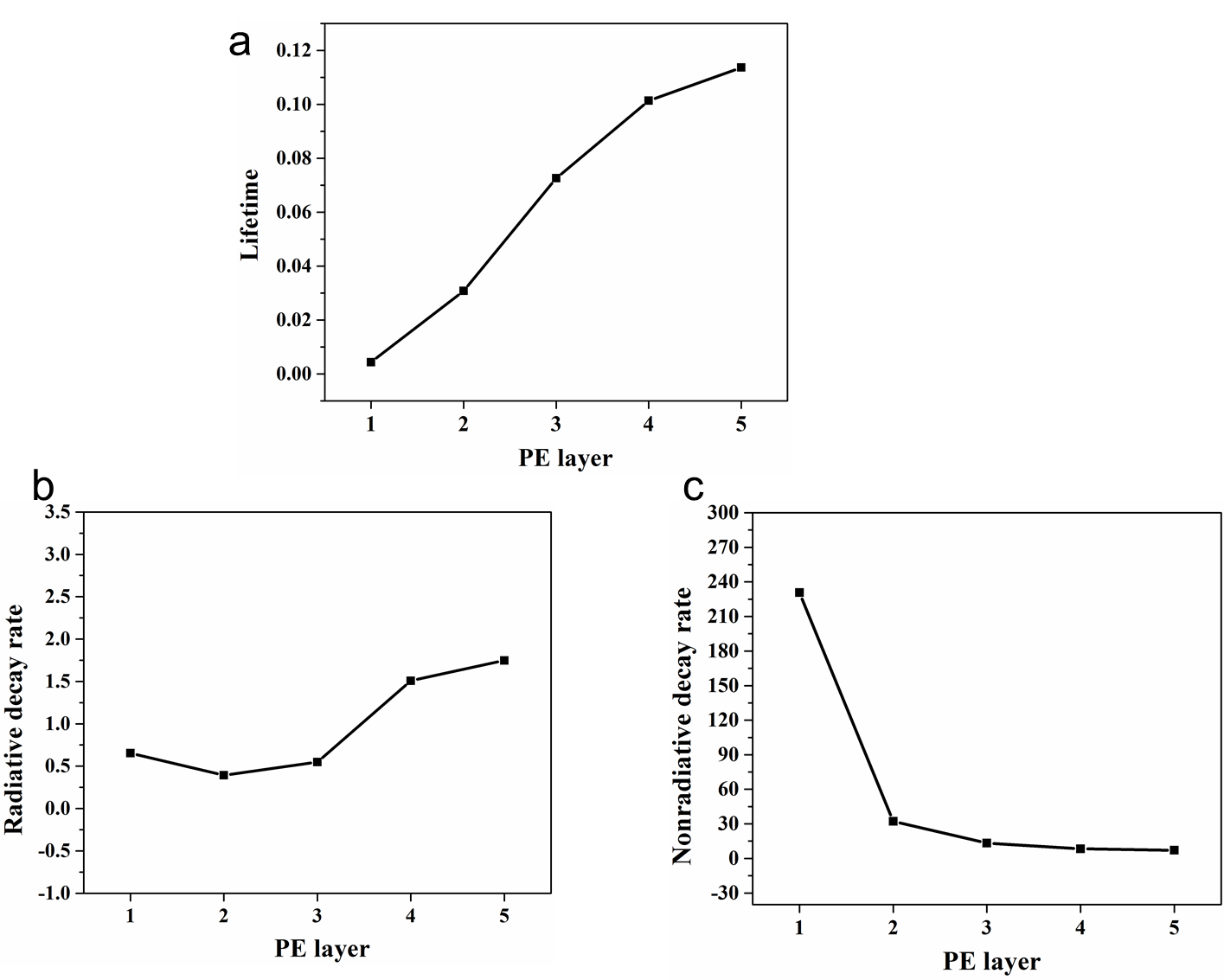


**Fig. S10.** (a) Lifetimes of excitons inside the plasmonic NCoM cavity at different positions. (b) Radiative decay rate and (c) nonradiative decay rate of the dipole of the plasmonic NCoM cavity at different positions.





**Fig. S11.** Radiation efficiency of the plasmonic nanocavity at different positions.

1. **The dark-field** **scattering spectrums study of plasmon-exciton system with different thickness of air spacers.**


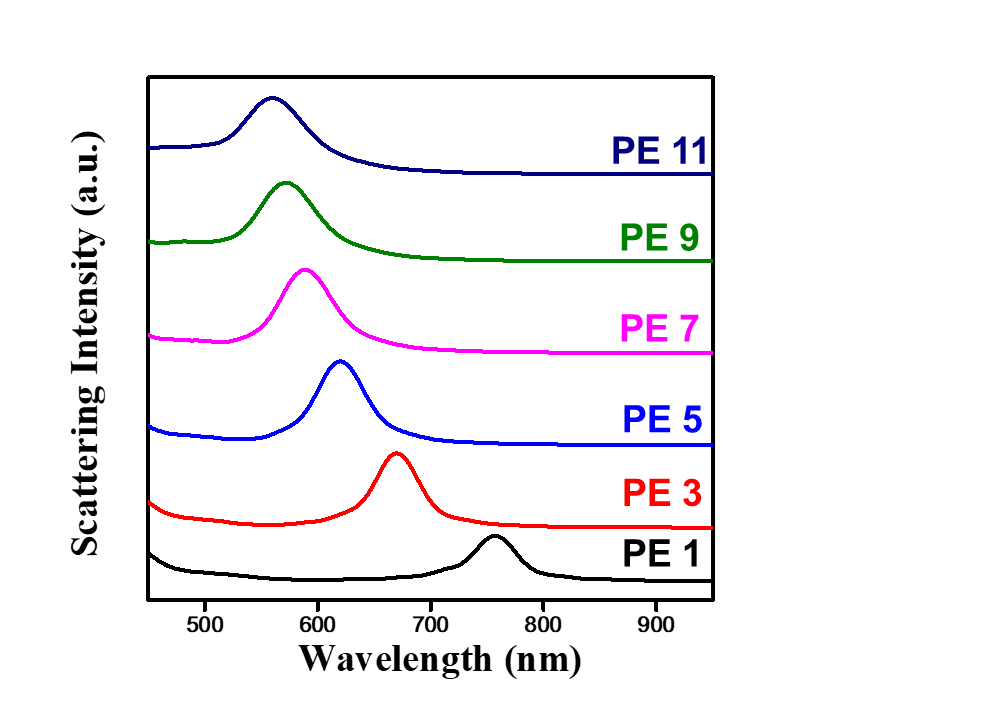


**Fig. S12.** The scattering spectrums of plasmon-exciton system with various thickness of air spacers.

1. L. Novotny, B. Hecht, Principles of Nano-Optics, Cambridge university press 2012.
